# Current Technologies of Electrochemical Immunosensors: Perspective on Signal Amplification

**DOI:** 10.3390/s18010207

**Published:** 2018-01-12

**Authors:** Il-Hoon Cho, Jongsung Lee, Jiyeon Kim, Min-soo Kang, Jean Kyung Paik, Seockmo Ku, Hyun-Mo Cho, Joseph Irudayaraj, Dong-Hyung Kim

**Affiliations:** 1 Department of Biomedical Laboratory Science, College of Health Science, Eulji University, Seongnam 13135, Korea; ihcho@eulji.ac.kr; 2 Department of Genetic Engineering, College of Biotechnology and Bioengineering, Sungkyunkwan University, Suwon City, Gyunggi Do 164-19, Korea; bioneer@skku.edu; 3 Department of Biomedical Laboratory Science, School of Medicine, Eulji University, Daejeon 34824, Korea; yeon@eulji.ac.kr; 4 Department of Medical IT Marketing, College of Health Industry, Eulji University, Seongnam 13135, Korea; mskang@eulji.ac.kr; 5 Department of Food and Nutrition, Eulji University, Seongnam 13135, Korea; jkpaik@eulji.ac.kr; 6 Fermentation Science Program, School of Agribusiness and Agriscience, College of Basic and Applied Sciences, Middle Tennessee State University, Murfreesboro, TN 37132, USA; seockmo.ku@mtsu.edu; 7 Korea Research Institute of Standards and Science, P.O. Box 102, Yuseong, Daejon 34113, Korea; hmcho@kriss.re.kr; 8 Department of Agricultural and Biological Engineering, Bindley Bioscience Center, Purdue Center for Cancer Research, Purdue University, 225 South University Street, West Lafayette, IN 47907, USA; josephi@purdue.edu

**Keywords:** electrochemical immunosensor, nanomaterials, point-of-care testing, signal amplification, electrode scaffold, labeling techniques

## Abstract

An electrochemical immunosensor employs antibodies as capture and detection means to produce electrical charges for the quantitative analysis of target molecules. This sensor type can be utilized as a miniaturized device for the detection of point-of-care testing (POCT). Achieving high-performance analysis regarding sensitivity has been one of the key issues with developing this type of biosensor system. Many modern nanotechnology efforts allowed for the development of innovative electrochemical biosensors with high sensitivity by employing various nanomaterials that facilitate the electron transfer and carrying capacity of signal tracers in combination with surface modification and bioconjugation techniques. In this review, we introduce novel nanomaterials (e.g., carbon nanotube, graphene, indium tin oxide, nanowire and metallic nanoparticles) in order to construct a high-performance electrode. Also, we describe how to increase the number of signal tracers by employing nanomaterials as carriers and making the polymeric enzyme complex associated with redox cycling for signal amplification. The pros and cons of each method are considered throughout this review. We expect that these reviewed strategies for signal enhancement will be applied to the next versions of lateral-flow paper chromatography and microfluidic immunosensor, which are considered the most practical POCT biosensor platforms.

## 1. Background

In 1962, Clark and Lyons introduced a glucose sensor that utilized a specific enzyme (i.e., glucose oxidase). The invention enabled a simple and on-site electrochemical analysis of blood glucose via the electrolysis of hydrogen peroxide produced from the enzyme-substrate reaction of the platinum electrode [1]. Thereafter, a variety of enzyme biosensors based on same principles have been consecutively proposed to measure various analytes—cholesterol, urea, ethanol, etc.—which allowed for the analysis of biological samples necessary for high-throughput and on-site evaluation. Enzyme-based electrochemical biosensors have recently been developed based on their response time, sensitivity and intrinsic selectivity [2] via cutting-edge nanotechnologies [3].

**Figure 1:**
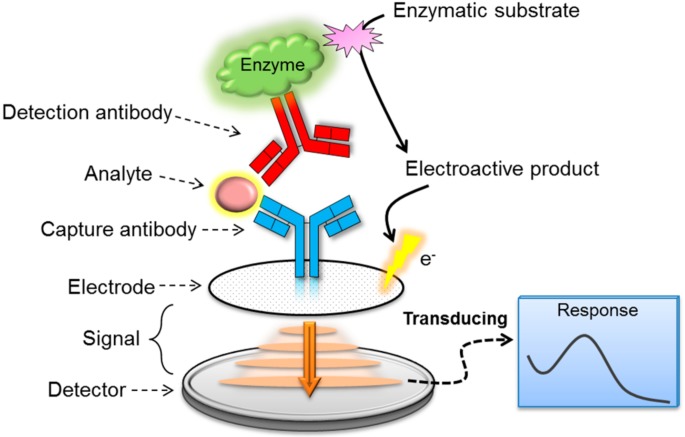
Basic analytical principle of electrochemical immunosensor.

The electrochemical immunosensor, a type of biosensor, employs the antibody as a capture agent and quantitatively measures the electrical signal resulting from the binding event between the antibody and target molecule (i.e., the analyte) as shown in Figure 1. This sandwich type of immunoassay, which is a common format of immunosensor, is based on the separation of unbound materials which do not participate in the binding event. The signals usually come from catalytic reaction of enzyme molecule labeled as a signal tracer with the detection antibody. The products containing electric charges can be detected by the electrode, thereby enabling a sensor device measurement for point-of-care testing (POCT). Different from the enzyme sensor, the immunosensor can be applied to a variety of sectors (e.g., medical, food industry, environments) since the antibody molecule is universal (compared to enzymes, which are limited to each substrate). The electrochemical immunosensor sensing principles can be categorized as follows: amperometry, potentiometry, conductometry and impedance [4,5], which depend on the measurement of specific signals.

There are many commercially-available immunochemistry analysis tools manufactured by the renowned diagnostic companies such as Roche, Abbott and Siemens. These models claim the highly sensitive measurement of target molecules (e.g., protein) up to 1–100 pg/mL in samples. However, these sophisticated clinical lab-scale instruments have limitations regarding their point-of-care regimes’ expensive reagents and bulky optical detectors. An electrochemistry-driven sensing platform can be used to develop the simple and miniaturized biosensors required for on-site analysis. However, developing an ideal POC immunosensor to comply with the sample rigor and detection limit still present a challenge. To this end, signal amplification strategies have gained increased attention for the high-performance analysis of the electrochemical immunoassay used for clinical diagnosis and environmental monitoring [6,7,8,9,10,11].

In this review, we, primarily focus on recently-developed strategies for signal amplification of electrochemical immunosensors’ ultrasensitive detection of target molecules. The amplification strategies are categorized into two sections. The first employs novel materials used as an electrode or supporting sensor matrix. The second includes various nanomaterial labeling approaches used as a carrier of electroactive tracers and enzymes and, performed by applying bioconjugation techniques. A large portion of these strategies have both paper-based and microfluidic-based immunosensor system applications, which are practical biosensor platforms for on-site biomolecule measurement.

## 2. Functional Nanomaterials Used as Electrodes and Supporting Matrices

**Figure 2:**
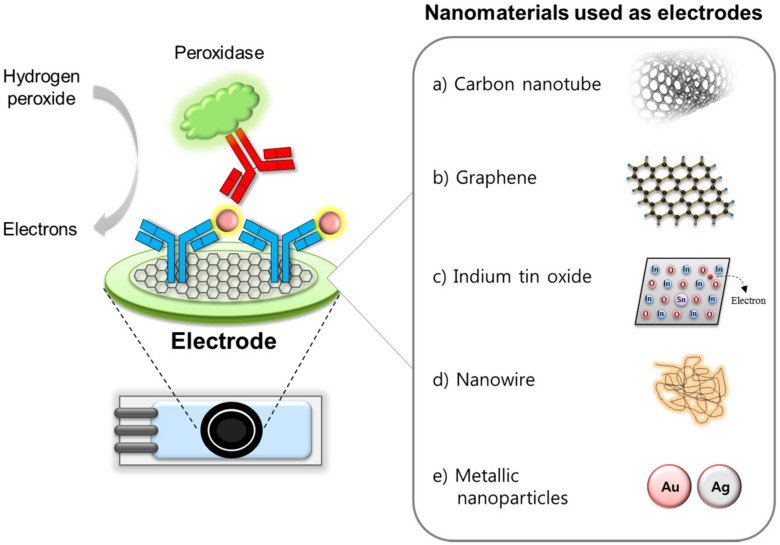
Nanomaterials used as electrodes or supporting solid matrices to enhance the analytical performance of electrochemical immunosensing.

The electrode in an electrochemical immunosensor provides a solid support for the immobilization of the capture binder (i.e., antibody) as well as a sensing means for the electrons produced from the biological reaction [12]. Therefore, choosing an appropriate electrode along with proper surface modifications is crucial due to several factors, in particular the analytical sensitivity. Many attempts have been made to improve the electrochemical properties of conventional and screen-printed carbon electrodes via employing various novel nanomaterials [13]. Such nanomaterials used as electrodes must meet the following requirements for enhancing electrical signals: sufficient electro-catalytic trait, adequate electron transfer ability and excellent biocompatibility with biological molecules such as capture antibodies [14]. Nanomaterials have huge surface areas, which can also support increased loading capacity and the mass transport of reaction molecules, resulting in a synergic contribution to signal amplification. This section will describe the select functional nanomaterials used as electrodes or supporting solid matrices as shown in Figure 2: the carbon nanotube [15,16], graphene [17], indium tin oxide [18,19], nanowire [6,20] and nanoparticles [21,22,23].

### 2.1. Carbon Nanotube and Graphene

Carbon nanotubes (CNTs) are extremely versatile and amenable to various scientific applications. CNTs can be used as electrode scaffolds in electrochemical sensing due to their outstanding chemical stability, mechanical properties, large surface area and conductivity resulting from enhanced the electron transfer reaction [15]. Antibody molecules can be immobilized on the CNT-modified electrode, where CNT surfaces are functionalized with amine- or carboxyl- groups [21]. There are two kinds of CNTs: single-walled carbon nanotubes (SWCNTs) and multi-walled carbon nanotubes (MWCNTs) as shown in Table 1 [16]. 

SWCNT characteristics can be described as follows. They feature a large surface area-to-volume ratio, low charge-carried density and delocalized π-orbitals [16]. Tlili et al. described an electrochemical immunosensor with high sensitivity and selectivity for alpha-amylase via the significant suppression of electrical noise using SWCNTs [16]. Here, the SWCNTs were used to bridge the lithographically patterned gold microelectrodes using AC dielectrophoresis. Powder SWCNTs have good dispersity, which enables uniform morphology and effective chitosan film. However, it is still challenging to use SWCNTs as electrodes in certain types of biosensors. For instance, Some biosensors have limited surface to interface with large biological recognition components such as mammalian cells, manipulate the sensor fabrication process, or undergo chemical functionalization [24]. To this end, more sophisticated sensors are necessary to address the issues related to blockage and the undesired interference caused by the nonspecific binding phenomenon which directly influences the selectivity and sensitivity of the sensor system.

MWCNTs that possess excellent conduction and electro-catalytic properties have also been utilized on the electrode as modified scaffold structures [15,25]. The surface can be modified for antibody immobilization via chemical treatment. Sánchez-Tirado et al. applied a copper-catalyzed azide-alkyne cyclo-addition to MWCNT surfaces to bind captured antibody via covalent linkage for cytokine detection (e.g., Transforming Growth Factor β1) [15]. Li et al. introduced the sulfonic acid group-functionalized MWCNTs as a supporting layer to increase the specific surface area as well as enhance the conductivity of glassy carbon electrodes. Gold nanoparticles were incorporated in this study for the purpose of improving not only the loading capacity of the capture antibody but also the acceleration of the electron transfer, therefore enabling the measurement of prostate-specific antigens ranging from 10 fg/mL to 50 ng/mL [22].

Graphene in the form of a two-dimensional honeycomb has gained considerable attention since its 2004 discovery. Graphene has been employed in many biosensor research fields, especially for electrochemistry-based sensing platforms. As shown in Table 1, graphene has the same intrinsic physical and chemical properties as graphite and CNTs, including a high surface area and numerous active sites. Graphene is superior to the other carbon allotropes based on the following properties: electron transfer speed, increased thermal conductivity, mechanical flexibility and biocompatibility [17]. However, graphene is hard to dissolve in water and thus its surface can be modified with hydrophilic functional groups such as the carboxyl group [26,27,28,29,30]. This process enables increased solubility as well as the higher efficiency of capture antibody immobilization via well-known amine-carboxyl chemistry assisted by 1-ethyl-3-(3-dimethylaminopropyl)carbodiimide (EDC).

Chen et al. used graphene nanocomposites to construct the electrochemical immunosensor and presented a concurrent analysis of multiplex cancer biomarkers [26]. Furthermore, combining gold nanoparticles and biopolymers with graphene-supporting electrodes allows captured antibodies to be immobilized and enables to consequently enable electron transfer to the electrode surface [26,31,32]. Reduced graphene (rGO) can also be employed as a supporting material for easier surface modification. Lai et al. proposed an ultrasensitive immunosensor based on electrochemical measurement of enzymatically-produced polyaniline [33]. In this study, aniline was polymerized via oxidation of the rGO electrode, to improve detection sensitivity within a wider dynamic range.

The use of carbon-based nanomaterials, e.g., CNTs and graphene, as supporting electrode matrices provide an appropriate environment to enhance electrochemical immunosensors signal. These nanomaterials feature several attractive properties: large surface area, superior electrical and thermal conductivity, high mechanical strength and high chemical stability. The incorporation of metallic nanoparticles further promotes electron transfer and the electrode’s signal susceptibility. However, the carbon-based nanomaterials have some limitations that must to be circumvented. 

One of the main problems with CNTs is that their processing is not fully controlled. Aggregation and low tube uniformity are examples. Furthermore, CNTs are commonly insoluble, which hinders their practical applications. This drawback can be overcome via the chemical modification of CNTs surface but it is not effective [34]. Compared with CNTs, graphene is a more attractive and versatile supporting matrix materials. However, improving its reproducibility and stability is a vital challenge for its high performance use [35]. With the more sophisticated process, graphene will broadly be utilized as an alternative means to the conventional electrode methods used in the electrochemical immunosensor.

### 2.2. Indium Tin Oxide

Indium tin oxide (ITO) shown in Table 1 has been widely used as an electrode due to its unique optoelectronic properties and high transmittance [18]. The advantages of ITO are its low cost, good electrical conductivity. The hydroxyl surface of ITO can be modified with various chemicals (e.g., silane compounds) to create functional layers terminated with amines, carboxylic acids, or thiols—often called the self-assembled monolayer (SAM)—for the immobilization of the capture antibody. This process constructs an electrochemical immunosensor. Bahadir et al. proposed a label-free ITO-based immunosensor for the detection of cancer biomarker C kinase I [19]. Here, the ITO-based substrate was chosen as a working electrode due to its high electrical conductivity, low detection limit (30 fg/mL) and wide dynamic range (14.25–712.5 fg/mL) of RACK1 in samples. 

Conducting polymer such as a polyaminobenzoic acid (PABA) can provide carboxyl groups on the ITO substrate and enable surface modification with active ester, which is highly reactive to the amine groups presented around an antibody molecule [36]. Increasing the size of the gold nanoparticle coupled with antibody detection via silver deposition induced the signal amplification of electrochemical impedance affected by the charge transfer to the ITO surface. Ion attachment is another signal enhancement strategy. Choi et al. proposed the polyvinyl-imidazole (PVI) polymer-modified ITO electrode with Ni(II) ions for the homogeneous measurement of hippuric acid known as a toluene metabolite [37]. Due to the high affinity trait of Ni(II) ions with the imidazole group, numerous Ni(II) ions bind to the polymer, thereby promoting electron transfer to the electrode. The electrical signals obtained from this technique showed a dynamic range between 0.1 µg/mL and 1.0 mg/mL proportional to the hippuric acid concentration. 

The ITO-based electrochemical immunosensor system can be utilized for the POC version due to its electrical conductivity and ease of applicability to be deposited as a thin film. Its glass-like characteristics allow for efficient antibody immobilization by forming different functional monolayers such as aldehyde, carboxylic acid, amine and sulfhydryl. Despite its extraordinary properties, the ITO-based electrode has some technical limitations as well. One of the main problems is that electron-transfer kinetics on ITO electrode is much slower than noble metal and carbon electrodes. This drawback intensifies when the electrodes are coated with biomolecules such as antibodies [38]. This phenomenon may be alleviated with proper surface modification of conducting polymers and electron mediators that facilitate electron transfer kinetics.

### 2.3. Nanowires

Nanowire materials have excellent potential as an alternative sensing strategy due to their small size, high surface-to-volume ratios and their electronic, optical and magnetic traits [20]. It is known that nanowire is more suitable and sensitive than larger bulky wires. Furthermore, its one-dimensional structure demonstrates high width-to-length ratio (1:1000 or higher), which results in unique physical properties similar to quantum phenomenon [24]. As shown in Table 1, electrical conductance of nanowires can be tuned by synthesizing different elements and chemical compounds such as metals (Ni, Cu, Au, Pt, etc.), metal oxides (ZnO, SnO_2_, Fe_2_O_3_), of semiconductors (Si, InP, GaN). Antibody molecules can also be immobilized on the nanowire for the construction of electrochemical immunosensors.

Silver nanowires have excellent electrical properties (e.g., rapid response, electro-catalytic capability and reproducibility) and can be employed as an efficient carrier of different signal tracers for various electrochemical measurements [6,39,40,41]. Cao et al. demonstrated interconnected silver nanowires to eliminate grain network randomization and to increase contact points for good electrical connection [6]. In this work, an electrical signal was generated by the reaction between horseradish peroxidase and hydrogen peroxide and enabled significant enhancement due to the superconductivity of the silver nanowires. This advantage featured a very low detection limit (4 pg/mL) with a broad range of linearity from 0.01 to 200 ng/mL. 

It also has been reported that some metal oxides such—zinc, iron, cerium, tin and titanium—exhibit facilitation of electron-transfer kinetics and influence the performance of nanowire-based electrochemical immunosensor. Furthermore, these metal composites make nanowire surfaces biocompatible and catalytic and improve their biosensing characteristics [42]. Wang et al. developed an TiO_2_ nanowire-assisted microelectrode-based immunosensor for the rapid detection of *Listeria monocytogenes,* which causes food poisoning outbreaks [43]. The diameter of nanowire was observed to be between 60 and 80 nm, where the captured antibody was immobilized. The impedance change caused by the nanowire antibody-bacteria complex was measured in proportion to the number of *Listeria monocytogenes*, resulting in the sensitive and rapid detection as low as 10^2^ cfu/mL of the bacteria within 1 h.

Semiconductor-based nanowire, e.g., silicon nanowire field-effect-transistors (Si-NWFETs), has been known as a promising electrical sensing platform due to its ultra-sensitive, real-time and label-free detection capabilities [44,45,46]. Kim et al. utilized a Si-NWFET- based immunosensor for the detection of cardiac troponin I, which is known as a specific biomarker of acute myocardial infarction [44]. Here, honeycomb-patterned nanowire was lightly doped on the FETs to enhance its sensitivity. The nanowire present superior detection limits as low as 5 pg/mL, which is considered a clinically meaningful value for the early diagnosis of acute myocardial infarction.

Nanowire conducting polymers can also be utilized for signal amplification in electrochemical immunosensor fabrication [47]. Hui et al. proposed electrochemically- synthesized polyaniline (PANI) nanowire for the measurement of alpha-fetoprotein, a biomarker of hepatocellular carcinoma [48]. PANI has usually been employed as an electron mediator to transport electrical signals toward the electrode, which can be facilitated by gold nanoparticles and enables not only conductance in neutral pH environments [48,49] but also increased charge transfer and PANI-based matrix stability [33,50,51,52]. Polyethylene glycol (PEG) was also employed as an additive to provide the electrochemical immunosensor with anti-fouling capabilities [48]. 

Although nanowire-based electrochemical measurement has several advantages over the described conventional approaches, a significant limitation is that the electrostatic potential of the nanowire that arises from the charge on the analyte molecule decays exponentially toward zero with distance (i.e., Debye length). To this end, a size reduction of the capture antibody by fragmentation with protease (e.g., Fab and F(ab’)_2_) or a single-chain variable fragment (scFv) offer technical solutions. The density control of capture antibody molecules onto the electrode surface can also be considered to reduce the intrinsic limitations of nanowire [53].

### 2.4. Metallic Nanoparticles

Metallic nanoparticles have been used as supporting materials that increase not only the efficiency of electron transfer but also the surface-to-volume ratio for the immobilization of capture antibodies, both of which significantly influence electrical signal enhancement as shown in Table 1 [21,22]. Gold nanoparticles can be employed to form electrodes due to their excellent electrochemical activity that comes from natural metallic trait where free electrons move from valence band to conduction band without an energy supply. Liu et al. claimed the highly sensitive detection of atrazine up to 0.016 ng/mL on the gold nanoparticle-modified electrode [23]. Other metallic nanoparticles (e.g., silver, copper, platinum, etc.) can be incorporated with various electrodes. Silver nanoparticles in particular have several advantages over others, namely their lower cost and higher conductivity. The use of metallic nanoparticles alone, however, is not sufficient for high-performance analysis, which eventually requires more elaborate approaches. Past work shows that electrical conductivity of reduced graphene oxide can be significantly enhanced by combining with silver nanoparticles and results from the correction of the structural defects of reduced graphene oxide [57]. Here the electrical conductivity of rGO/AgNPs composites was significantly improved compared to rGO alone by an enhancement factor of 346% with AgNPs-assisted compensation of the rGO structural defect. This reflects the importance of metallic nanoparticles used as supporting materials in electrochemical immunosensors.

Recently, hybrid electrodes based on gold nanoparticles and associated with other materials such as silicon oxide [58], carbon nanosphere [59] and calcium carbonate [60], have been introduced to improve the synergistic properties affecting analytical performance. For instance, gold nanoparticles were chemically deposited on thiolated reduced graphene oxide film onto the surface of a screen-printed carbon electrode for the detection of p53 antigen, which resulted in high sensitivity (0.088 pg/mL of the limit of detection) [56]. Li et al. proposed a core-shell nanocomposite that consists of amino-functionalized cuprous oxide and ceric dioxide (Cu_2_O@CeO_2_-NH_2_) to physically connect with the gold nanoparticles [13]. Due to the excellent electro-catalytic properties of cuprous oxide nanocrystals and, the reduction of hydrogen peroxide and free radical scavenging properties of cerium dioxide [61,62,63,64], this sensing system showed a very low detection limit of 0.03 pg/mL, which enabled the highly-sensitive analysis of prostate specific antigen (PSA) under non-label conditions.

However, electrical instability is one of the disadvantages of metallic nanoparticles because of its susceptibility to salt concentrations which may induce aggregation to be precipitated. Therefore, the proper chemical and biological modifications of the nanoparticle surface are crucial to the utility of these nanoparticles in human samples with high salt concentration. Moreover, metallic nanoparticle-driven signals are inconsistent upon signal amplification, which lowers reproducibility [17]. Therefore, quality control of nanoparticle prepared via liquid-phase reaction in combination with a fine-tuning reduction process should be considered for better performance.

## 3. Signal Enhancement via Labeling Techniques

**Figure 3:**
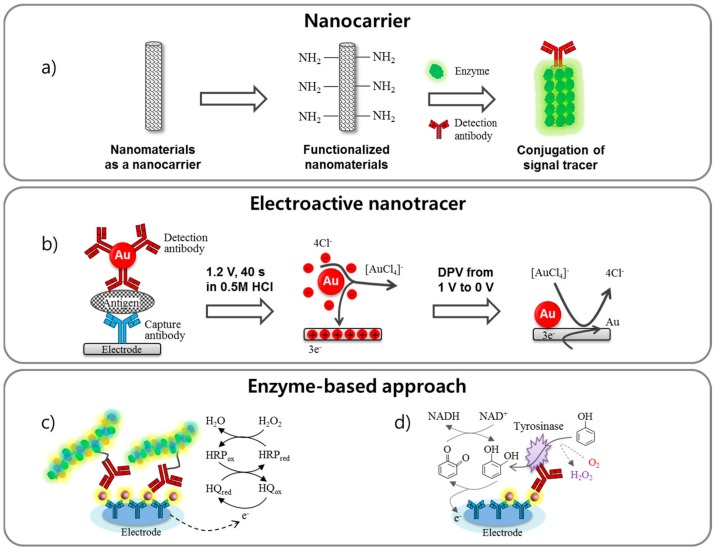
Schematic representation of labeling approaches for the signal amplification of electrochemical immunosensor. The labeling techniques can be categorized as (**a**) nanocarrier, (**b**) electroactive nanotracer, (**c**) enzyme-based nanopolymer and (**d**) redox cycling of enzyme reactions, which enable an electrochemical signal enhancement.

Signal amplification via sandwich-type electrochemical immunoassay can also be achieved by labeling tracers such as enzyme and nanoparticles with detection antibodies. Although the molecular orientation of antibodies on the surface is a core strategy of signal amplification, a labeling approach based on various bioconjugation techniques associated with nanomaterials can have a greater influence on increased sensitivity. As shown in Figure 3, these strategies can be categorized as follows: (a) loading numerous electroactive species and amplifying the electrical signal using nanomaterials as carriers [65,66,67]; (b) using metal and semiconductor nanoparticles as electroactive labels [67,68,69,70]; (c) utilizing enzyme-functionalized nanopolymers. (d) Also, redox cycling can be a part of signal enhancement strategies by applying reducing agents to efficiently convert the oxidized state of a species to the reduced state [71]. Accordingly, this section will describe the signal amplification based on the labeling methods.

### 3.1. Nanocarriers

Since nanomaterials (NMs) have a large surface area that influences the loading capacity of signal tracers such as enzymes and electroactive compounds, NMs can act as ideal nanocarriers in electrochemical immunosensors as shown in Table 2 [72,73]. These include gold nanoparticles, magnetic beads, single- and multi-walled carbon nanotubes, silica nanoparticles, graphene oxides, dendrimers and electroactive component-encapsulated nanoparticles. 

Recently, the mesoporous silica nanoparticle (MSN) has widely been used as a nanocarrier due to its high surface area, tunable pore structure and modifiable surface [74,75]. Fan et al. proposed a MSN-based controlled release system with acid cleavable linkage for quantitative analysis of the prostate specific antigen [76]. In this study, a thionine electron mediator was encapsulated by capping the MSN pores with carboxylic acid modified gold nanoparticles, which could be removed under acidic conditions. The process resulted in the release of thionine. This study exhibited a low limit of detection (0.31 pg/mL) and a wide dynamic range (0.001–50 ng/mL). 

Graphene and graphene oxide (GO) also have higher loading capacities compared to nanoparticles, which leads to the use of graphene nanosheets as a carrier. Du et al. proposed a functionalized GO as a carrying body of multi-enzymes for the ultra-sensitive detection of phosphorylated p53 (Ser392), which is known as a tumor suppressor and transcription factor [77]. This approach was achieved by linking horseradish peroxidase and a p53^392^—specific antibody to the GO at a high ratio, therefore amplifying electrocatalytic response with the reduction of enzymatically-oxidized thionine in the presence of hydrogen peroxide. Incorporating nanoparticles can also be a good nanocarrier. Zhong et al. proposed a graphene nanocomposite decorated with gold nanoparticles and doped with an ionic liquid, which was used to immobilize alkaline phosphatase (ALP) and antibody labeled with ferrocene [78]. Due to the high loading capacity of ALP as well as the facilitation of the electron transfer, the sensitivity was significantly enhanced and exhibited, exhibiting a very low detection limit of 40 fg/mL with a dynamic range of 0.1–80 pg/mL. 

Nanocarriers that bear a high capacity of signal molecules due to their large surface area allow a dramatic increase in the production of electrochemical signals from the immunoreaction. This approach is a very effective way to improve the analytical performance of the biosensor system without additional surface modifications such as patterning or sputtering methods which are regarded as complicated, laborious and costly. However, most of the nanomaterial construction and conjugate processes associated with signal tracers were not completely established regarding uniformity, distribution, shape and molar ratio, which are critical factors that need to be considered upon labeling. Therefore, an improved protocol for the preparation of a unique nanocarrier, along with the proper conjugation strategy, is required for improved performance. Also, the diffusional limitation inside the nanocarrier may be a potential problem, especially in the case of using enzymes. Here, a hydrodynamic layer formed by water molecules usually affect the phenomenon, which interferes with the accessibility of the substrate to the immobilized enzyme in the carrier. Therefore, the proper design and distribution of enzyme molecules in the carrier is a key consideration upon preparing the conjugation.

### 3.2. Electroactive Nanotracer

Nanomaterials, particularly metal nanoparticles (e.g., colloidal gold and silver) have been used as electroactive nanotracers, along with functional electrodes, in the construction of efficient electrochemical immunosensors [67,70] as shown in Table 2. The nanoparticle is usually coupled with the detection antibody, enabling the production of electrochemical signals based on the redox properties of the nanoparticles in acidic condition [79]. Here, gold nanoparticles can be reduced under the pre-oxidation process of high potential at 1.2 V for 40 s, quantitatively measuring human chorionic gonadotropin (hCG) [79] by cyclic voltammetry. The results showed a linear relationship between the reduction peak and hcG concentration (0–500 pg/mL) with a LOD of 5 pg/mL. Gold nanoparticles have a catalytic characteristic that facilitates chemical reaction such as the reduction of silver ions to metallic that can be deposited onto the gold nanoparticles. This deposited silver layer can be converted into silver ions via electrochemical oxidation, which can be quantitatively measured in proportion to analyte concentration [80,81].

Compared to the nanoparticles in the colloidal range, the smaller nanogold (1–2 nm in diameter) showed significant potential as an electroactive material due to its superior catalytic activity and colloidal range. Lin et al. designed nanogold-functionalized mesoporous carbon foam (Au/MCF) to support the synergistic silver enhancement process of carbon-gold [82]. Nanogold underwent in situ growth on carboxylated MCF, which was then coupled with the detection antibody. High signal detection sensitivity can be achieved by electrochemical stripping of the deposited silver, which led wide linear dynamic range of 50 fg/mL to 1 ng/mL along with 24 fg/mL LOD. 

Likewise, silver nanoparticles have been utilized as an electroactive nanotracer because it can be oxidized at a more negative potential and produce a sharper peak compared with gold nanoparticles [72]. This nanomaterial can also alleviate the background signals observed in gold nanoparticles under extremely acidic conditions [83]. Yin et al. employed silver nanoparticles coated with silicon dioxide (Ag@SiO_2_) as a signal amplification unit for the following reasons: easy dissolution in nitrate, simple synthesis, easy surface modification and good biocompatibility [84]. Under optimal conditions, they claimed a high-performance analysis associated with a wide dynamic range (0.2 to 500 nM) and low detection limit (78 pM). 

Unlike colloidal particles, Pd-based bimetallic nanostructures (e.g., Pd–Pt, Au–Pd, Pd–Co., Pd–Fe) recently showed distinguished performance in electrochemical detection. This advantage is caused by the fact that these materials have extraordinary properties: enhanced catalytic capability, adsorption and charge transfer traits [85,86,87,88]. For an example, Au-Pd nanocrystals loaded on boron-nitride nanosheets presented good electrocatalytic activity and long-term stability, high conductivity and biocompatibility [89]. Using the nanohybrid structure, Sharma et al. demonstrated the ultrasensitive detection of a *Bacillus anthracis* biomarker with a detection limit of 1 pg/mL. 

This approach, however, is limited in that the electroactive nanotracer requires additional reaction steps for electrical signal generation under extremely low pH solution. Due to harsh reaction conditions, this method may not be compatible with a POCT version of the platform, especially lateral-flow immunochromatography.

### 3.3. Enzyme-Based Approach

Due to its excellent catalytic activity and substrate specificity, enzymes have been widely employed as a tracer via detection antibody labeling in combination with electron mediators in common electrochemical enzyme immunoassays. As shown in Table 2, simply increasing the number of enzyme molecules that form a polymeric enzyme has been the main strategy to enhance analytical sensitivity. One of the methods is to couple several enzymes to a detection antibody using branch-like functionalized structures (e.g., amine-terminated dextran). Xiong et al. proposed an antibody-enzyme network structure composed of a dextran-amine skeleton anchoring more than 100 and 15 molecules of HRP and secondary antibody, respectively [90]. This allowed many enzyme molecules to participate in the binding event, resulting in signal enhancement significant—enough to detect a target analyte that exists in extremely low concentrations.

This type of enzyme-based signal amplification can be further improved by modulating the redox cycling associated with electron mediators such as ferrocene [91] and hydroquinone [90] as shown in Table 2. Ferrocene transfers one electron to the electrode by a simple redox reaction. This approach can be achieved by using a reducing agent to convert the oxidized signal species state to the reduced state. As an example, a chitosan-ferrocene electrode composited with gold nanoparticles provided an excellent electrode surface and served as an ideal electrochemical indicator of the immunoassay process detection of carcino-embryonic antigen [91]. Also, Akanda et al. presented a novel tyrosinase-responsive redox cycling [71]. In the study, the low electro-activity of the substrate and high oxidation of the NADH reducing agent potential can resulted in a negligible background. However, NADH-related electrochemical catalysis upon the quick formation of the highly electro-active enzymatic product significantly increased the voltammetric response, which allowed a high signal-to-noise ratio. 

Although enzyme-based reactions have several advantages in terms of signal generation (usability, high specificity and sensitivity as described elsewhere), this approach also features the following weakness, as a tracer in electrochemical immunoassay: (i) relatively narrow dynamic range, (ii) lengthy incubation time required to obtain a detectable signal and (iii) sample matrix susceptibility, which may contain analytes that interfere with the enzyme. Hence, establishing an optimal condition for the enzyme-substrate reaction is crucial. Reducing a diffusional limitation of the substrate is a possible approach. Also, removing or alleviating all possible inhibitors in the sample that may influence the enzyme reaction should be considered.

## 4. Summary and Outlook

Nanotechnology efforts have developed innovative electrochemical immunosensors by signal amplification strategies for the highly-sensitive analysis of point-of-care testing. Accordingly, novel nanomaterials (e.g., carbon nanotube, graphene, indium tin oxide, nanowire and metallic nanoparticles) are usually employed to construct high-performance electrode-supporting materials due to their high conductivity, high surface areas, etc. The surfaces of these nanomaterials can thus be modified with a variety of organic layers—silanes, thiols and conducting polymers—for efficient immobilization of biomolecules to impact specific functions. In this review, we also discussed alternative ways to enhance electrochemical signal sensitivity by introducing labeling technologies applied to a number of electroactive, electroactive nanotracers and signal-generating species via enzyme catalysis of polymeric enzyme and redox cycling. These approaches can be applied to the POCT-version of electrochemical systems exemplified by lateral-flow immunochromatography and microfluidic devices through screen-printing, patterning and carrying electroactive species, which both miniaturized and improved the analytical performance. Future studies will further develop this advanced technology. Although the electrochemical immunosensor has proven propitious for sensitive detection in clinical and environmental applications, the matrix interference from real samples (e.g., blood, foods, etc.) is still one of the critical issues that needs to be circumvented in order to improve specificity and stability of sensor systems.

## Figures and Tables

**Table 1:** Summary of representative nanomaterials used as electrode scaffolds for signal enhancement. Abbreviation: Single-wall carbon nanotube (SWCNT); Multi-wall carbon nanotube (MWCNT); Indium tin oxide (ITO); Nanowire field effect transistors (NWFETs); cardiac troponin I (cTnI).

Materials	Examples	Advantages	Limitations	Limit of Detection	Linear Range	Ref.
**(a) Carbon-based**	SWCNT	Large surface area to volume ratio (S/V) Low charge-carried Density Delocalized π-orbitals	Difficult manipulation during sensor fabrication process Difficult chemical functionalization	hCG (2.4 pg/mL)	10–2000 pg/mL	[54]
	MWCNT	Excellent conducting and electro-catalytic properties	Need to functionalize surface for increasing biocompatibility	PSA (3.33 fg/mL)	10 fg/mL–100 ng/mL	[22]
	Graphene	High S/V Large active sites Fast electron transfer High thermal Conductivity Better mechanical Flexibility Good biocompatibility	Hard to dissolve in water	CEA (0.10 pg/mL)	0.01 pg/mL–1.0 ng/mL	[55]
**(b) ITO**		Low cost/High Transmittance Good electrical Conductivity Ease of surface Modification	Slow kinetics of electron-transfer upon coating surface with antibodies	RACK1 (30 fg/mL)	14.25–712.5 fg/mL	[19]
**(c) Nanowire**	Metal	Rapid response, electro-catalytic capability and reproducibility	Decrease in electrostatic potential with distance	IgG (4 pg/mL)	0.01–200 ng/mL	[6]
	Metal oxides	Facilitation of electron-transfer kinetics	The same as above	*Listeria Monocytogenes* (10^2^ cfu/mL)	No linear range can be found	[43]
	Semi-conductor	Ultrasensitive/Real-time Label-free in NWFETs	The same as above	cTnI (5 pg/mL)	5–200 pg/mL	[44]
	Conducting polymers	Maintenance of conductance under neutral pH Improvement of the charge transfer and stability	The same as above	AFP (7 fg/mL)	0.01 pg/mL–1.0 ng/mL	[48]
**(d) Metallic nanoparticle**	Au, Ag, composites	Efficient electron Transfer Increase in S/V Supplying superior conductivity	Electrical instability in high salt concentration Inconsistent upon signal amplification	Atrazine (16 pg/mL) p53 (88 fg/mL) PSA (30 fg/mL)	0.05–0.5 ng/mL 0.1 pg/mL–10 ng/mL 0.1 pg/mL–100 ng/mL	[23] [56] [13]

**Table 2:** Summary of labeling strategies associated with nanomaterials for signal enhancement. Abbreviation: Mesoporous silica nanoparticle (MSN); Graphene oxide (GO); Alkaline phosphatase (ALP); Prostate-specific antigen (PSA); Apyrimidinic endonuclease (APE); Human chorionic gonadotropin (hcG); Carcinoembryonic antigen (CEA); Alpha-fetoprotein (AFP).

Strategies	Example	Effects	Limit of Detection	Linear Range	Ref.
**(a) Nanocarrier**	MSN	Encapsulation of electron mediator	PSA (0.31 pg/mL)	0.001–5.0 ng/mL	[76]
	GO	High loading capacity of ALP	Human apurinic/APE 1 (40 fg/mL)	0.1–80 pg/mL	[78]
**(b) Electroactive nanotracer**	Colloidal gold	Redox properties in acidic condition Facilitation of chemical reaction	hCG (5 pg/mL)	0–500 pg/mL	[79]
	Nanogold	Superior catalytic activity to colloidal gold	CEA (24 fg/mL)	0.05 pg/mL–1.0 ng/mL	[82]
	Silver nanoparticle	Production of sharper peak compared to gold nanoparticle	*N*6-methyladenosine (78 pM)	0.2–500 nM	[84]
	Bimetallic nanostructures	Enhanced catalytic capability Excellent adsorption and charge transfer trait	*Bacillus anthracis* (1 pg/mL)	5 pg/mL–100 ng/mL	[85]
**(c) Enzyme-based approach**	Antibody-enzyme network structure	Increasing the number of enzyme molecules	AFP (2 pg/mL)	5–200 pg/mL	[90]
**(d) Redox cycling**	Facilitation by electron mediators	Converting the oxidized state of signal species with reducing agents	CEA (sub pg/mL)	1.0 pg/mL–0.1 μg/mL	[71]
